# Exaggerations and Caveats in Press Releases and Health-Related Science News

**DOI:** 10.1371/journal.pone.0168217

**Published:** 2016-12-15

**Authors:** Petroc Sumner, Solveiga Vivian-Griffiths, Jacky Boivin, Andrew Williams, Lewis Bott, Rachel Adams, Christos A. Venetis, Leanne Whelan, Bethan Hughes, Christopher D. Chambers

**Affiliations:** 1 Cardiff University Brain Research Imaging Centre (CUBRIC), School of Psychology, Cardiff University, Cardiff, United Kingdom; 2 School of Psychology, Cardiff University, Cardiff, United Kingdom; 3 School of Journalism, Media & Cultural Studies, Cardiff University, Cardiff, United Kingdom; 4 School of Women’s and Children’s Health, University of New South Wales (UNSW), Kensington, New South Wales, Australia; 5 Graduate School of Medicine, Faculty of Science, Medicine and Health, University of Wollongong, Wollongong, Australia; University of Sheffield, UNITED KINGDOM

## Abstract

**Background:**

Exaggerated or simplistic news is often blamed for adversely influencing public health. However, recent findings suggested many exaggerations were already present in university press releases, which scientists approve. Surprisingly, these exaggerations were not associated with more news coverage. Here we test whether these two controversial results also arise in press releases from prominent science and medical journals. We then investigate the influence of mitigating caveats in press releases, to test assumptions that caveats harm news interest or are ignored.

**Methods and Findings:**

Using quantitative content analysis, we analyzed press releases (N = 534) on biomedical and health-related science issued by leading peer-reviewed journals. We similarly analysed the associated peer-reviewed papers (N = 534) and news stories (N = 582). Main outcome measures were advice to readers and causal statements drawn from correlational research. Exaggerations in press releases predicted exaggerations in news (odds ratios 2.4 and 10.9, 95% CIs 1.3 to 4.5 and 3.9 to 30.1) but were not associated with increased news coverage, consistent with previous findings. Combining datasets from universities and journals (996 press releases, 1250 news), we found that when caveats appeared in press releases there was no reduction in journalistic uptake, but there was a clear increase in caveats in news (odds ratios 9.6 and 9.5 for caveats for advice and causal claims, CIs 4.1 to 24.3 and 6.0 to 15.2). The main study limitation is its retrospective correlational nature.

**Conclusions:**

For health and science news directly inspired by press releases, the main source of both exaggerations and caveats appears to be the press release itself. However we find no evidence that exaggerations increase, or caveats decrease, the likelihood of news coverage. These findings should be encouraging for press officers and scientists who wish to minimise exaggeration and include caveats in their press releases.

## Introduction

Press releases have long been used as 'information subsidies' to facilitate science news [1], and have become the dominant link between academia and the media [2,3]. As structural changes to the news industry’s revenues and profitability have put pressure on staffing levels, journalists are expected to produce more copy in less time. Due to these economic contexts, journalists in general, and science/medical journalists in particular, routinely use the content of press releases in their news stories, often without sufficient checks and research to independently evaluate the claims [4–9]. Since the majority of people, at least in western populations, use news media as their main source for up-to-date science and health information [10], science press releases could have a large cumulative effect on public perceptions of science and on health-related behaviours [8,11–16].

Press releases routinely condense complex scientific findings and theories into digestible packets, providing an efficient means for disseminating new science of interest to publics in a helpful form for journalists [17]. Information and quotes in a press release are highly likely to be included in related news [18]. However, because of this synergy, any exaggerations, message creep or misinformation in press releases may also be reproduced in the news [19,20]. On the other hand, caveats to strong or simplistic claims in press releases are rarely present [20], presumably because they are assumed to hamper a clear news message and therefore to harm news interest and/or be excluded from news anyway.

In a study of health-related news and press releases based on research in 20 major UK universities, we previously found that common types of subtle exaggeration frequently appearing in news were highly associated with what was written in the corresponding press release [20]. We analysed advice given to readers, causal claims based on observational data and claims about humans based on non-human research. We found that a third or more press releases contained stronger advice, causal statements or human claims than any found in the peer-reviewed journal article they were based on. Moreover, the existence of these forms of exaggeration in press releases strongly predicted their presence within news (odds ratios between 6.5 and 56). Counter to common assumption, however, exaggerated press releases were not more likely to attract news. In other words, the main source of news exaggerations appeared to be not the journalists themselves, but the text of academic press releases. But the premise upon which academics might be tempted to subtly exaggerate in their press releases—that it would encourage more news uptake—appeared to be false.

While university press releases have a clearly defined role in facilitating the communication of science, they are just one source of science news. Previous research has indicated that press releases distributed by academic journals are potentially more important [7,9]. If the associations between news and journal press releases are similar to those with university press releases, this will show the provocative results of our previous study hold in distinct population. If the results show a different pattern from those with university press releases, then any effort to address how exaggerations appear in health and science news will have to differ for the different pathways.

There are several reasons why university press releases and journal press releases might differ with regard to subtle exaggerations and their transmission into news: in universities and academic institutions, press officers routinely involve academic authors in the development of press releases, whereas not all journals do this (partly due to volume and time pressures), and academic authors may have less expectation that they will be heavily involved (the journal normally owns the copyright). Relatedly, journal press offices may have stronger reliance on the text of the peer-reviewed paper, and may also have access to the academic editor for the paper. In the case of non-human studies, a journal may have less reason to worry about drawing attention to animal research facilities as they are unlikely to suffer reputational or material damage from the release of such information in the same way a university might. Other environmental and stylistic differences exist as well, but there is, to date, no directly comparable data to assess rates of exaggeration and news uptake (e.g. [21–23]).

The scientific process relies on not just making conclusions, but explaining why such conclusions can be made, whether strong or weak conclusions are justified and what the limits of each conclusion should be (caveats). Interviews with press officers [17] suggest it should be common to find such caveats and explanations at the end of press releases, because they are important information for news consumers with illnesses or considering changes to health-related behaviour. But caveats (e.g. '*We still need more research to clarify whether vitamin D directly prevents bowel cancer or if people with higher levels are generally healthier')* or justifications for the strength of conclusions (e.g. ' *Unlike previous researchers [authors] looked at children from all social backgrounds')* appeared in only about 10% of press releases and news in Sumner et al. (2014) (see also [22]). It is commonly assumed that caveats would harm news interest or would be excluded by journalists even if included in press releases. In the second part of this study we assess whether these assumptions hold, or whether caveats might in fact be beneficially included in press releases. We similarly analyse the use of justifications in the translation of nuanced scientific findings into clear news stories. These questions require a very large dataset.

In sum, this study first investigates whether exaggerations in science and health news are associated with exaggerations in journal press releases, and whether press releases that were exaggerated attracted more news than those that did not. We analysed the text of press releases from major journals, the text of the news arising from these press releases, and the text in the associated peer-reviewed papers. Secondly, we analyse the use and effects of caveats and justifications by combining new data from journal press releases with previously collected data from university press releases [20].

## Methods

Press releases based on possible relevance to human health, psychology or neuroscience were identified for studies published in the following journals, for the entire year of 2011: Lancet, British Medical Journal (BMJ), Science, Nature, Nature Neuroscience, Nature Immunology, Nature Medicine, and Nature Genetics. The press releases were collected from either publicly available repositories (journal web pages or EurekAlert) or press sites for science journalists (Nature Publishing Group kindly provided us with free access to all their press releases for the purpose of this study). This resulted in 534 relevant press releases with associated peer-reviewed journal articles. News articles (n = 582) resulting from these press releases were identified from UK national news media by searching the Nexis database, BBC.co.uk, uk.reuters.com, and by performing a Google search.

The process of data extraction and analysis was identical to that in Sumner et al. (2014). Research assistants recorded by hand specific information about the statements and other content of press releases, peer-reviewed papers and news articles according to a set list of 191 questions in the coding sheet (available in the open data depository, see below; many of these questions did not apply to every press release, depending on content). The coding sheet focused on advice given, causal claims, and conclusions about humans based on non-human research, as explained further below. Statements and information were assessed first in press releases and news stories, and then compared to corresponding information and statements in the peer-reviewed journal articles, which were always taken as baseline.

### Health advice

Each source was coded for maximum level of advice out of four possible categories: no advice, implicit advice (e.g. 'for adults with a BMI greater than 35 kg/m2…permanent calorie reductions of more than 500 calories per day would be needed…'), explicit advice but not to the reader or general public (e.g. 'clinicians must exercise caution in their use of aflibercept'), or explicit advice to the reader or general public (e.g. 'patients who are at increased risk of cardiovascular events should consider starting statin treatment promptly and continuing it long-term'). The set of journal article, press release and news was included in the analysis if at least one source contained advice that was implicit or explicit; there were 247 such sets, with 411 news stories.

### Causal claims

Press releases, news articles and journal articles were coded for the strength of the main statement of findings (according to 7 levels: no statement, statement of no relationship, correlational, ambiguous, conditional causal, can cause, and causal) in either the title and the first two sentences of the news and the press releases, or abstract and discussion in the journal articles. We used the first two sentences of news and press releases because they follow a structure where the main claims are stated first, and these are likely to have the largest influence on readers. Thus the question is about the main statements, not what is stated in supporting text further down the articles. This analysis focused on studies based on correlational cross-sectional and longitudinal designs, and excluded all qualitative, intervention, or simulation designs. In total 164 press releases and associated journal articles and 237 news stories were included in the analysis.

### Human inferences from research on non-humans

For each journal article based on a non-human sample (animals, cells, or simulations), the associated press release and news article were coded for whether the findings or conclusions were stated as explicitly non-human, implicitly human or explicitly human. There were 112 journal articles and press releases and 64 news stories. However, we could not do most of the analyses for this type of exaggeration since only one exaggerated press release had associated news. Therefore raw results are in supporting information (S1 File).

### Caveats and Justifications

Because caveats and justifications tend to be rare, in order to analyse them we combined datasets from the journal press releases described above with our previous study of university communications (Sumner et al., 2014), which used identical procedures. The presence, or not, of caveats for causal/correlational statements were coded for every relevant press release (N = 428); caveats for explicit advice could be coded only where explicit advice appeared (N = 188). Presence or absence of justifications for causal statements were coded if the press release contained any explicit statement of relationship (N = 355), while justifications for advice were coded only for those that contained explicit advice (N = 188).

### Double coding

The coding for each journal article, press release, and associated news stories took 3–4 hours per set; the entire dataset required approximately 700 full days of research time before analysis. Within this, we randomly selected 147 press releases (27.5% of all press release sample) and associated 144 news stories (24.7% of all news sample) for independent double-coding (i.e. a different research assistant coded the entire set again), which resulted in concordance rate of 98.3% for the results reported below (κ = 0.97). Note that where concordance is high and resource limitations require a direct choice between more double coding or collecting more samples (in this case increasing the N for press releases and news stories), statistically the latter strategy is better for the reliability of results.

### Analysis

As in our previous study, exaggeration was defined as claims or advice in press releases or news that were coded at a level above any of the associated statements in the peer-reviewed paper. For example, where the news makes a causal claim but the journal article did not. Thus we are recording what might be termed message creep beyond the journal article text; we are not, in this research, attempting to judge whether the journal article itself contains exaggeration, as there would not be universal agreement on what was scientifically accurate; [24]. Therefore our definition of 'exaggeration' means 'exaggeration beyond any that might already exist in the peer-reviewed paper'.

For direct comparison to Sumner et al (2014), we used generalised estimating equations [25] to determine percentages and 95% confidence intervals (CI) for exaggeration rates, while adjusting for the clustering of several news articles per press release (using an exchangeable working correlation). The generalised estimating equations framework was also employed to estimate the association (in odds ratios) between exaggeration in the press release and exaggeration in the news, and the association between caveats or justifications in press releases and news. Note that these analyses included only those journal articles and press releases for which there was at least one news story (and the news could be appropriately coded for the relevant analysis). We compare news uptake (press releases with and without associated news) using bootstrapped 95% CI and standard inferential statistical tests, since there is no clustering issue for these analyses.

All coding sheets (N = 534 for journal press releases, N = 462 for university press releases), full instructions for coding, and data analysis files and programs are available online at https://github.com/SolveigaVG/JournalPROpenData.git and http://dx.doi.org/10.6084/m9.figshare.903704

## Results

### 1. Exaggeration rates in press releases

We found that 23% of press releases in the advice analysis (56/247; Table 1) included more direct or explicit advice than the associated journal article (bootstrapped 95% confidence interval, CI, 17% to 28%). Similarly, 21% of press releases about correlational research (35/164; Table 1) contained exaggerated causal statements (CI 15% to 27%). These rates are not negligible, but are both lower than those we previously measured in equivalent analyses for university press releases, which were 40% (CI 33% to 46%) and 33% (CI 26% to 40%), respectively. For research on non-humans, we present partial results in section A of supporting information (S1 File); we could not do all analyses due to low N. It is worth bearing in mind that our definition of exaggeration was claims that go beyond those in the peer-reviewed paper. If we had the expertise to judge exaggerations against the results of each paper rather than the statements within those papers, then we would likely find many papers already containing exaggeration and thus total exaggeration rates would be higher than our results depict.

**Table 1:** Summary of key results.

	N	PR with news	N news	Odds news uptake	Odds ratio (95% CI)	Odds news exaggerated	Odds ratio (95% CI)
**Advice**	247	120	411				
PR not exaggerated	191	101	354	1.1	0.5 (0.2–0.8)	0.3	2.4 (1.3–4.5)
PRs exaggerated	56	19	57	0.5		0.8	
**Causal claims**	164	86	233				
PR not exaggerated	129	63	166	1	1.9 (0.9–4.8)	0.4	11 (3.9–30)
PRs exaggerated	35	23	67	1.9		4.1	

Further information, including percentages and 95% CIs, are provided in text and Figs. Partial results for non-human studies are in supporting information because low N meant some analyses could not be performed.

### 2. Association between press release and news exaggeration

**Fig 1:**
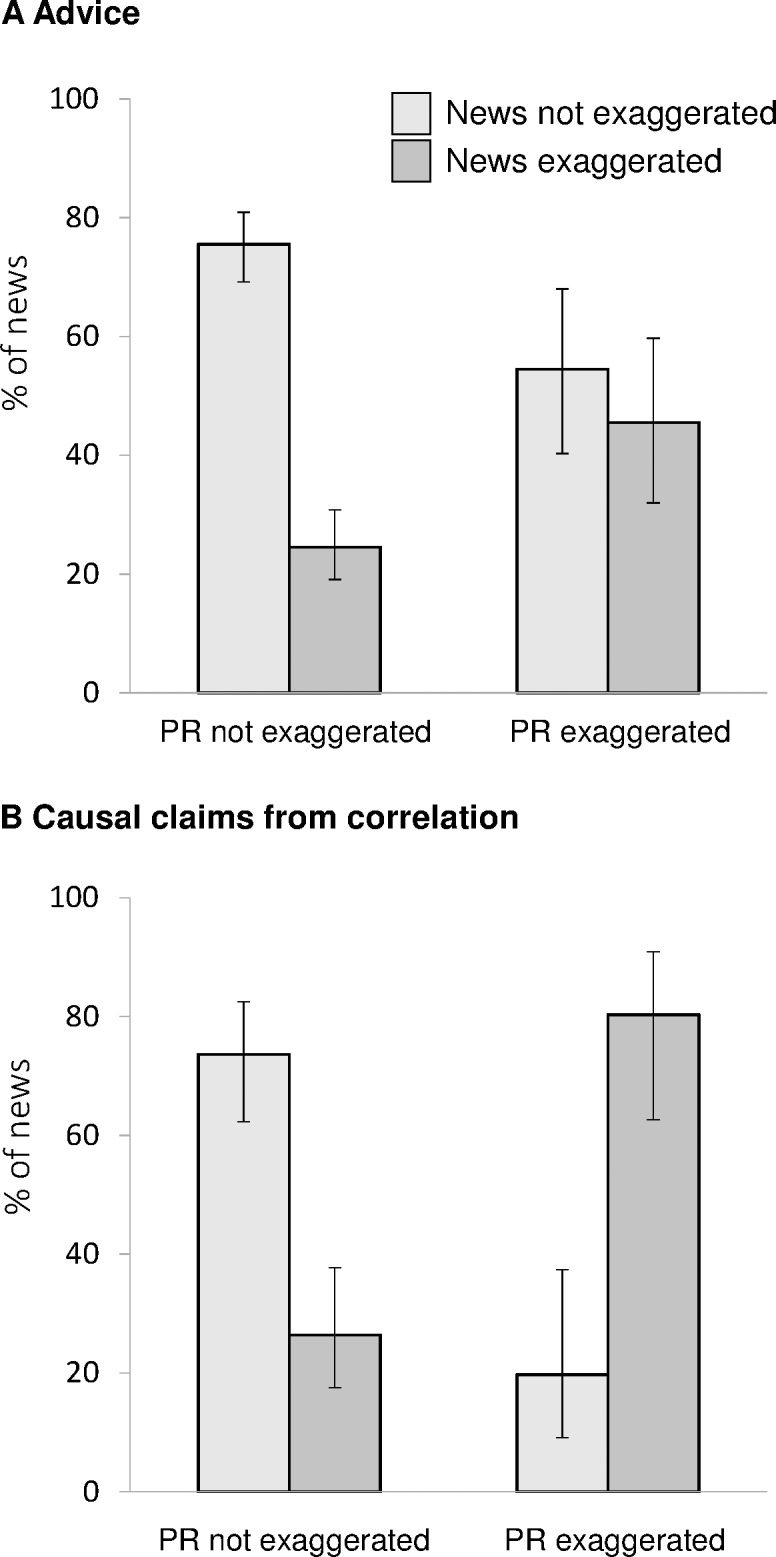
Association between press release and news exaggeration. The proportions of news with exaggerated advice (A), or causal statements from correlational research (B) were higher when the associated press releases (PR) contained such exaggeration (N for Advice, PR = 247, news = 411; causal claims, PR = 164, news = 237). Error bars are 95% confidence intervals. See Table 1 for odds ratios. Partial results for non-human studies are in supporting information because low N meant this analysis could not be performed.

As shown in Fig 1, there was strong association between exaggerations in press releases and news. For advice, 27% of news (CI 22% to 33%) had more direct or explicit advice than the journal article, and the odds of this were 2.4 times higher (odds ratio 2.4, CI 1.3 to 4.5) when the press release also contained exaggerated advice (46%, CI 32% to 60%) than when it did not (25%, CI 19% to 31%). For main statements describing correlational studies, 38% of news stories (CI 27% to 51%) had stronger causal wording than the associated journal article, and the odds of exaggeration were 10.9 times higher (CI 3.9 to 30.1) when press releases contained exaggerated causal claims (80%, CI 63% to 91%) than when they did not (26%, CI 18% to 38%). This pattern is highly similar to that found for university press releases (cf Fig 2 in Sumner et al. 2014).

### 3. No association between press release exaggeration and news uptake

**Fig 2:**
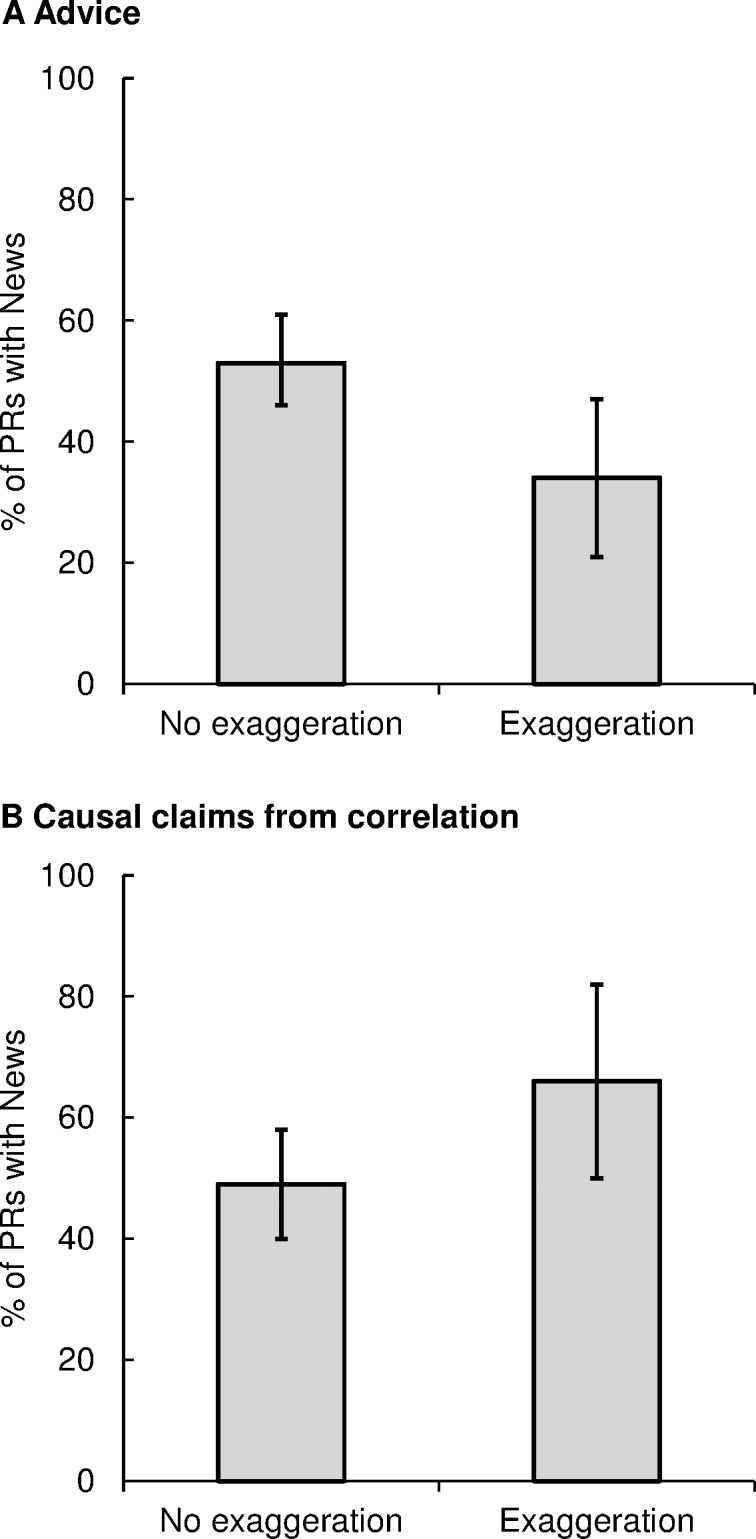
No Effect of press release exaggeration on news uptake. The proportion of press releases (PRs) that have resulting news articles when the press releases do not contain exaggerations (left bars) compared to when they do (right bars) for analyses of advice (A) and causal claims from correlation (B). Error bars are bootstrapped 95% confidence intervals. See Table 1 for odds ratios. Note that full analysis for non-human studies could not be performed because only one exaggerated press release had associated news.

There was no evidence that exaggeration in press releases is associated with increased news uptake (Fig 2). For advice, 101/191 (53%) press releases without exaggeration had news articles while only 19/56 (34%) of press releases with exaggeration had news (difference -19%, CI -5% to -34%). For causal claims, 63/129 (49%) of press releases without exaggeration had news compared with 23/35 (66%) of press releases with exaggeration (difference 17%, CI -1% to 35%).

Similarly, of those press releases that had some news, the number of news stories was not greater for press releases with exaggeration. When press releases did not contain exaggerated advice, they were associated with 3.5 news stories per press release, compared to 3.0 news stories per press release that did contain exaggerated advice (CI of difference -1.5 to 0.6). Press releases not containing exaggerated causal claims were associated with 3.1 news stories per press release, compared to 3.0 news stories per press release containing exaggerated causal claims (CI of the difference -1.2 to 1.2). This lack of association between exaggeration and news uptake echoes the results for university press releases (cf Fig 3 in Sumner et al., 2014).

**Fig 3:**
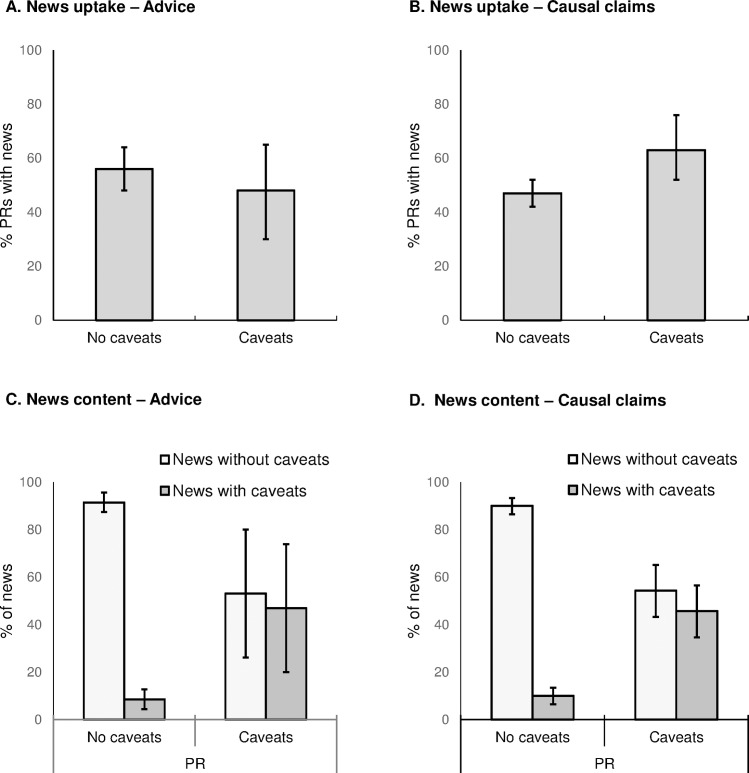
Press release caveats, news uptake and caveats in news. (A) News uptake for press releases (PRs) with and without caveats for explicit advice. (B) News uptake for PRs with and without caveats for causal claims. (C) Association between caveats for explicit advice in the PR and caveats for explicit advice in resulting news articles. (D) Association between caveats for causal claims in the PR and caveats for causal claims in resulting news articles. All error bars are bootstrapped 95% confidence intervals.

### 4. Caveats for advice and causal claims

The use of caveats is of key interest for understanding the difficult translation of nuanced scientific findings into clear news stories. Caveats were rare in university press releases (Sumner et al. 2014), which might reflect the assumption that they hamper a clear message and thus potentially harm news interest, or the belief that they would be ignored by journalists and are therefore a pointless addition to the press release. This rarity has previously limited any systematic analysis. Here, we combined the data for journal press releases with the previously published dataset for university press releases (Sumner et al. 2014), given that the main results reported above were similar across both sources of science news. The overall rate of caveats in press releases were 15% (29/188) for advice and 14% (62/428) for causal statements about correlational research.

We found no evidence that caveats reduce news uptake. For advice there was news uptake for 48% (14/29) of press releases with caveats, and similarly for 56% (89/159) of press releases without caveats (CI of the difference: -28% to 12%; see Fig 3A and 3B). For causal statements there was news uptake for 63% (39/62) of press releases with caveats, compared to 47% (171/366) without caveats (CI of the difference 3% to 29%). Thus, if anything, caveats for causal statements are associated with greater news uptake, although we cannot say whether this is in any part due to the caveat itself, or additional characteristics of press releases that also include caveats.

We found caveats do get into news. Caveats for advice appeared in 37/264 (14%) of news stories, and caveats for causal statements appeared in 107/607 (18%) of news. This low rate, taken alone, might imply that journalists tend to avoid caveats, but when compared to the caveats in press releases a different picture emerges (Fig 3C and 3D). The odds for caveated advice were 9.5 times higher (odds ratio 9.5, CI 2.8 to 32) when the press release contained caveats (47%, CI 20% to 74%) than when it did not (8.5%, CI 4.4% to 13%). The odds for caveated causal statements were 7.6 times higher (odds ratio 7.6, CI 4.2 to 14) when the press release contained a caveat (46%, CI 35% to 57%) than when it did not (10%, CI 6.5% to 13%). Thus caveats in press releases do not appear to be routinely ignored by journalists. We also found no association between caveats and exaggeration within press releases or news (S1 File, section B). In other words, caveats were not systematically used to balance exaggeration, and neither were they systematically associated with an overall cautious (non-exaggerating) approach.

### 5. Justifications for advice and causal claims

The overall rates of justifications in press releases were 10% (19/188) for advice and 21% (74/355) for causal statements about correlational research.

**Fig 4:**
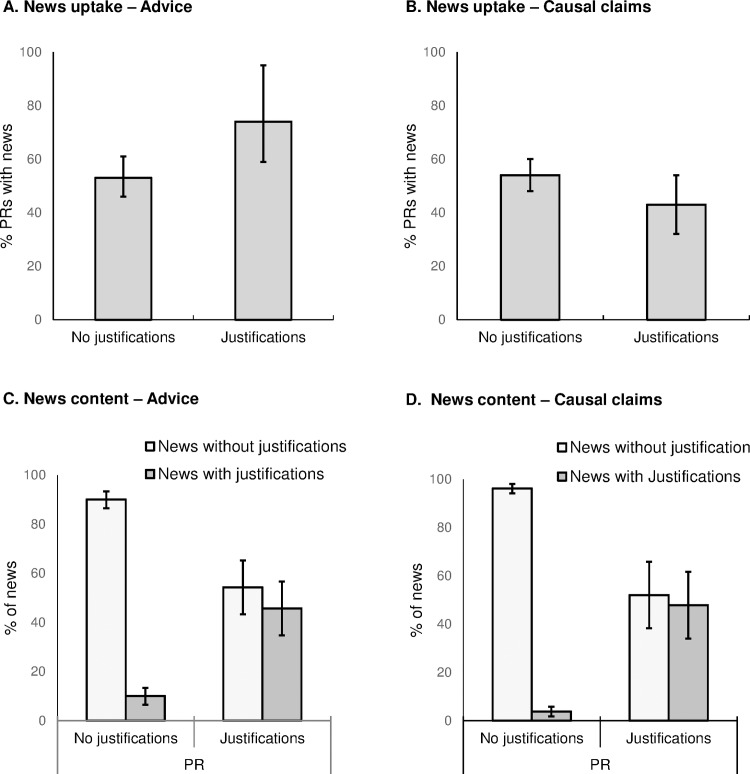
Press release justifications, news uptake and justifications in news. (A) News uptake for press releases (PRs) with and without justifications for explicit advice. (B) News uptake for PRs with and without justifications for statements of relationship. (C) Association between justifications for explicit advice in the PR and justifications for explicit advice in resulting news articles. (D) Association between justifications for statements of relationship in the PR and justifications for statements of relationship in resulting news articles. All error bars are bootstrapped 95% confidence intervals.

We found no clear relationship between justifications and news uptake. For advice there was news uptake for 74% (14/19) of press releases with justifications, and for 53% (89/169) of press releases without justifications (bootstrapped CI of the difference: -2% to 41%). For causal statements there was news uptake for 43% (32/74) of press releases with justifications, and 54% (151/281) without justifications (CI of the difference -15% to 2%; see Fig 4A and 4B)).

We found justifications are reported in news. Justifications for advice and causal statements appeared in 41/264 (16%) and 69/538 (13%) of news stories, respectively. The odds for advice were 6.1 times higher (odds ratio 6.1, CI 2.0 to 18) when the press release contained justifications (42%, CI 19% to 66%) than when it did not (11%, CI 5.5% to 16%). The odds for causal statements were 23 times higher (odds ratio 23, 95% CI 11 to 50) when a press release contained justifications (48%, CI 34% to 64%) than when it did not (3.8%, CI 1.8% to 5.8%; Fig 4C and 4D). Thus, like caveats, justifications appear to be noticed and utilised. We also found that justifications were included more often alongside exaggerated advice, but for causal claims were instead associated with a more cautious approach in which exaggeration is diminished in news (S1 File, section C).

## Discussion

In summary, we obtained the following key results: 1) Exaggeration rates were lower in our sample of journal press releases compared to the previous study of university press releases; 2) Exaggerations in news were strongly associated with exaggerations in press releases; 3) Exaggerations were not associated with higher likelihood of news or more news stories; 4) Caveats in press releases did not seem to harm news uptake, and were strongly associated with higher likelihood of caveats appearing in associated news pieces; 5) Similarly, justifications in press releases were associated with higher likelihood of similar statements in news, but had no consistent effect on news uptake. Results 2 and 3 generalise (to a new and even larger dataset) the provocative findings of a previous study (Sumner et al. 2014). Result 4 challenges received opinion that explicit caveats hamper newsworthiness and/or are likely to be ignored by journalists.

Overall then, we find strong associations between press releases and news for all the types of phrases and sentences analysed (exaggerations, caveats or justifications), but no evidence that such factors systematically influence whether a press release is judged newsworthy. The relationships between press release content and news apply to both the major sources of science news: universities and journals. These results should be encouraging to press release authors (press officers and scientists) who would prefer to take a more nuanced approach but have feared losing news interest.

Note that the types of exaggeration studied here are quite subtle and routine: for example changing a phrase like 'related to' or 'might increase' to a direct causal phrase like 'boosts'. We have no evidence that these small exaggerations (or message creep) represent conscious efforts to hype, and interviews with press officers show they feel a strong obligation to avoid hype [17]. Unintended subtle exaggerations may arise for other reasons such as trying to use simpler and more direct language with fewer words per sentence. Moreover, within our data will be cases (especially in some examples of health advice given to readers) where exaggeration as defined here could be considered entirely appropriate rephrasing within the context of the press officers' professional vision [17]. In the other direction, our data doubtless include cases where strong statements–statements that would be considered hype by some readers–were not defined as exaggeration because they were already contained in the peer-reviewed journal article, which we employed as the best available baseline. If we had the expertise to judge exaggerations against the results of each paper rather than the statements within those papers—or were able to assess consensus opinion from experts in each field about hype in each paper—then we would likely find many papers already contained exaggeration and thus total exaggeration rates would be higher than our results depict. These inevitable variations and contextual factors in individual cases are why it was important to generate very large dataset–the largest of its kind ever studied–to extract overall patterns, and to base conclusions on those patterns rather than exact percentages of exaggeration.

Although exaggerations in science and health reporting are often subtle, we do believe that the cumulative effect of hundreds per year might be damaging to a reader's understanding of health-related matters, and to their understanding of the relative uncertainty of different conclusions, such as those based on observational research vs randomised controlled trials. Recent data shows that observational studies are just as likely as RCTs to receive news coverage [26]. It is also possible that trust in medical research may be eroded when, over time, opposing conclusions are both presented with apparent high certainty, or seemingly conflicting advice is offered to readers. Erosion of understanding and trust in scientific research and advice could then hamper public health initiatives such as changing alcohol guidelines, and they may have large knock on effects for high profile controversies, such as have arisen over vaccines or statins.

One limitation of this work is the number of journals sampled, which was set primarily to achieve a manageable project; we also note that not all journals issue press releases (e.g. the *New England Journal of Medicine*). The major limitation of this work is its observational (correlational) nature. It is always possible that some of the apparently causal links are circumstantial or mediated by factors we did not analyse. For example the rare use of caveats might potentially have co-occurred with unknown other characteristics influencing news uptake in order to disguise any caveat influence. The possibility of unknown correlated factors is generally true for correlational research.

We also cannot be sure that the relationships between press releases and news reported here would continue to hold as strongly if circumstances changed. For example, if the rates of caveats were to increase markedly, it is possible that they would be ignored much more often; their current impact on news might be related to their low frequency. It is only possible to definitively answer such questions by experimentally changing the current rate.

Exaggerations in journal press releases were lower than in the university press releases studied previously [20]. This is not consistent with the assumption held by many scientists that it is academic authors who reduce exaggeration in press releases; authors tend to be less involved in writing and revising journal press releases than university press releases. Instead, the different rates may reflect the different professional habits, contexts, priorities, and pressures of press officers working in each environment. For instance there are fewer elite scientific/medical journals than universities, and because their publication dates are regular, they are an established feature of journalistic routines (news diaries) in all national and specialist news outlets. Thus perceived competition between journals is likely to be less marked than between universities.

Exaggerations and caveats for human inference drawn from non-human research were especially low in number, which meant we could not meaningfully analyse them further. However the low numbers are interesting in themselves. Firstly, exaggeration rates were much lower than for university press releases (11%, CI 5% to 17%, vs 36%, CI 28% to 46%), which we speculate may reflect journals worrying less than universities about revealing animal research programs and thereby attracting the attention of protestors or suffering reputational damage. It will be interesting to discover whether the Concordat on Openness on Animal Research changes current practice in this regard. Secondly, caveats for non-human research were very low across both datasets (N<5), even compared to the low rate of caveats overall. Our study offers no explanation for this trend.

In conclusion, the data presented here indicate that it might be possible to avoid some common small exaggerations and to increase the use of caveats and justifications in press releases without hampering news uptake. The evidence also suggests that these small changes to press releases are likely to carry through into news. This might be one important part of the challenge to enhance understanding and reduce confusion about lifestyle choices relevant to health.

## Supporting Information

S1 File Supporting information. (PDF)
