# Supplementary material for: Exaggerations and Caveats in Press Releases and Health-Related Science News
Source: PLoS One. 2016 Dec 15;11(12):e0168217. doi: 10.1371/journal.pone.0168217 (PMC5158314; doi:10.1371/journal.pone.0168217)
Supplement: S1 File — (PDF) [file pone.0168217.s001.pdf]

Exaggerations and caveats in press releases and health-related science news

Supporting Information

A. Human claims from non-human studies.

For each journal article based on a non-human sample (animals, cells, or simulations), the associated press release and news article were coded for whether the findings or conclusions were stated as explicitly non-human, implicitly human or explicitly human. There were 112 journal articles and press releases and 64 news stories that contained such sample information.

Exaggeration rates. 11% of press releases about non-human studies made inflated inferences about humans (CI 5% to 17%). 27% (CI 4% to 49%) of news articles based on non-human studies contained inflated human inference. While the proportion of exaggeration in news for non-exaggerated press releases is similar to that for advice and causal statements, it was not possible to analyse the association between exaggeration in news and the press release because only one exaggerated press release generated news.

News uptake: For human inference, 27/100 (27%) of press releases without exaggeration had news compared to 1/12 (8%) press releases that had exaggerated human inference (-19% difference, CI -24% to 2%). When press releases did not have exaggerated human inference, they were associated with 2.2 news stories per press release compared to 2.0 news stories per press release that had exaggerated human inference (CI of the difference not possible due to N=1 in exaggerated PR group).

Caveats and Justifications. For caveats and justifications about human inference about animal research, there were also too few to analyse (N<5) despite combining datasets from journal and university press releases.

Table S1. Summary of results for our analyses of human inference from non-human studies ('human inference'). While N are low for some bins, these results, as far as they go, appear in line with those presented in the main paper for exaggerations in advice and causal claims.

	N	PR with news	N news	Odds news uptake	Odds ratio (95% CI)	Odds news exaggerated	Odds ratio (95% CI)
**Human inference**	112	28	56				
PR not exaggerated	100	27	54	0.4	0.3	0.4	Not possible
PRs exaggerated	12	1	2	0.1	(N too small)	N too small	

Supporting Information

B. No association between caveats and exaggeration.

Caveats were not associated with exaggeration within the same press release or news. Caveats did not appear to be generally used to redress the balance following exaggerated advice or causal statements in the same text. On the other hand, neither did they appear associated with a generally cautious approach in which exaggeration is less likely. For advice, 41% (12/29) of press releases with caveats also had exaggeration, while a similar 46% (73/159) without caveats had exaggeration (95% CIs of the difference -16% to 24%). Similarly in news, 76% (28/37) with caveats also had inflation, and 67% (152/227) without caveats had inflation (95% CIs on the difference -7% to 23%). For causal statements, the pattern was similar: 19% (12/62) of press releases with caveats had exaggeration, and 18% (52/293) without caveats contained exaggeration (95% CIs of the difference -8% to 15%). Similarly, 36% (38/107) of news with caveats had exaggeration, and 32% (139/431) without caveats contained exaggeration (95% CIs of the difference -4% to 17%).

Caveats do not appear to reduce news exaggerations. In news stories arising from press releases containing caveats for advice, 52% (24/46) contained inflated advice, while only 32% (90/278) contained inflated advice when the press release had no caveat (95% CIs of the difference 4% to 35%, p=0.006). Similarly, for causal statements, news stories arising from press releases with caveats were more likely to have exaggeration (55/132, 42%) than news stories from press releases without caveats (122/477, 26%; 95% CIs on the difference 3% to 22%, p=0.004; the analyses here and below employed the condensed causal coding described in section 4 above, but the same pattern was obtained using the original method, see Figure 4). While it is possible that caveats increase the likelihood of exaggerations, there may be other differences between these groups of press releases that account for this relationship. For example, caveats might be included in press releases when the scientist or press officer regards the likelihood of news exaggerations to be high.

Supporting Information

C. Association between justifications and exaggeration.

Justifications in news were associated with increased advice but less exaggerated causal statements. Justifying advice was associated with more explicit advice than in the journal article, while justifying causal statements appears to be associated with a generally more cautious approach with less exaggeration. These patterns in news were not so evident in the press releases. For advice, 58% (11/19) of press releases with justifications also had exaggeration, while 44% (74/169) without justifications had exaggeration (95% CIs of the difference -10% to 38%). However in news, 93% (38/41) with justifications also had exaggeration, while only 64% (142/223) without justifications had exaggeration (95% CIs on the difference 18% to 38%). For causal statements, 22% (16/74) of press releases with justifications had exaggeration, and 17% (48/281) without justifications contained exaggeration (95% CIs of the difference -6% to 17%). However in news, only 19% (13/69) of news with justifications had exaggeration, while 35% (164/469) without justifications contained exaggeration (95% CIs of the difference 3% to 14%,).

Justifications may increase infated advice in news. In news arising from press releases that provided justifications for advice, 52% (30/58) contained inflated advice, while only 32% (84/266) contained inflated advice when the press release had no justification (95% CIs of the difference 6% to 34%). Note that although we code the advice as inflated because it did not appear in the journal article, it may be appropriate advice and explicit justification may help journalists to make this judgment. However, for causal statements, news stories arising from press releases with justifications were not more likely to have exaggeration (37/129, 29%) than news stories from press releases without justifications (124/400, 31%; 95% CIs on the difference -6% to 10%).
